# Measurement of Knowledge and Practice of Saudi Population Towards Hernias and Its Risk Factors

**DOI:** 10.7759/cureus.33264

**Published:** 2023-01-02

**Authors:** Sultan Alsaigh, Shatha Alotaibi, Renad Aljasser, Shahd AlMarei, Shahad Alahmadi, Abdullah Madkhali, Ibtihal Balubaid, Abdulrahman Hakami

**Affiliations:** 1 General Surgery, King Fahad Specialist Hospital, Buraidah, SAU; 2 College of Medicine, Qassim University, Unaizah, SAU; 3 College of Medicine, Qassim University, Buraidah, SAU; 4 College of Medicine, Jazan University, Jazan, SAU; 5 College of Medicine, AlRayyan Colleges, Madinah, SAU; 6 College of Medicine, King Abdulaziz University, Jeddah, SAU; 7 Medicine Department, College of Medicine, Jazan University, Jazan, SAU

**Keywords:** inguinal, ksa, risk factors, hernia, adults

## Abstract

Background: A hernia is an aponeurotic defect that allows an organ to protrude from its normal cavity. Despite advancements in hernia care, hernia patients' experiences with care, as well as recurrence and complication rates, are frequently suboptimal. Adequate knowledge of the risk factors of a hernia could lead to a significant reduction in the prevalence of hernia. Therefore, the current study aimed to assess the awareness of the risk factors of abdominal hernias among adults in Saudi Arabia.

Methodology: Our study is a cross-sectional analytic study to measure the level of knowledge, awareness, and practice of ventral and inguinal hernias in Saudi Arabia. The data was collected by using a valid pretested structured questionnaire taken from previous studies after getting writing approval.

Results: In this study, we were able to collect data from 2611 individuals in different regions of Saudi Arabia, where 68.5% of the participants were females and 60.1% were aged between 18-29 years old. The prevalence of hernia among individuals in the current study was 9.2%, associated with participants older than 40 years old (19.1%), participants of the northern region (16.6%), illiterate individuals (30.0%), married (13.2%), and overweight or obese individuals (10.9% and 12.6%). In general, 53.7 % of the participants had a moderate level of knowledge, while 23.8% had a high level and 22.5% had a low level of knowledge.

Conclusion: We found a moderate level of knowledge among adult individuals in Saudi Arabia about hernia. The prevalence of hernia was similar to those reported in previous studies; however, there is a higher incidence of risk factors in the current population.

## Introduction

A hernia is an aponeurotic defect that allows an organ to protrude from its normal cavity. Hernia diagnosis and management have clearly developed since the earliest recorded case of an inguinal hernia around 1500 BC. The discovery of essential anatomic elements that are specific to each hernia type has allowed surgeons to develop a variety of technical approaches that result in more efficient repairs. The pre-operative and post-operative care of these patients has also improved. Despite advancements in hernia care, hernia patients' experiences with care, as well as recurrence and complication rates, are frequently suboptimal [1].

Although such hernias can be small and asymptomatic, the majority of them cause pain and suffering to patients, lowering their quality of life. Furthermore, a small percentage of hernias lead to incarceration and even strangulation of the bowel and other viscera, which can be fatal. When you consider that 1 in 5 individuals who have a laparotomy will develop an incisional hernia, it's evident that abdominal wall hernias are still a prevalent and serious healthcare issue. Abdominal wall hernia (AWH) is one of the most crucial for all surgeons who operate on the abdomen [2].

Ventral/incisional and inguinal hernias are two of the most common surgical conditions that surgeons treat. Inguinal herniorrhaphies are the most common general surgical procedure, with over 2800 cases per million population in the United States and Europe. In the United States alone, about 800,000 inguinal hernia repairs are performed each year. Over 400,000 operations are performed each year for ventral/incisional herniorrhaphies, with a projected increase of 11,000 cases per year. Re-operations for recurrences with estimated ranges of 24-43 percent and 1-15 percent for ventral and inguinal hernia repairs, respectively, are included in these figures. Despite the use of mesh in 70-85 percent of inguinal and 85.8% of ventral hernia repairs, these recurrences nevertheless occur [1]. Ventral/incisional and inguinal hernias are two of the most common surgical conditions that surgeons treat. Inguinal herniorrhaphies are the most common general surgical procedure, with over 2800 cases per million population in the United States and Europe. In the United States alone, about 800,000 inguinal hernia repairs are performed each year [1].

The Prevalence of Hernia Repair and its Associated Risk Factors in Saudi Arabia was studied in 2018, and the results revealed that 3.8% of the participants had had hernia repair [3]. In terms of risk factors that might lead to a hernia, 29.1% of the participants had a positive family history of hernia repair surgery, 44.5% were obese, 27.6% were smokers, and 25.8% ate a high-fat diet. 17.5% had chronic constipation, 12.6% had a chronic cough, 8.3% had major surgeries, 7.7% were diabetic, 5.2% had been admitted to the intensive care unit, 4.6% had urinary retention, 2.1% had a major trauma, 1.25% had been diagnosed with atherosclerosis disease, and 37.7% had no risk factors [3]. The main objective of this study is to assess the awareness of the risk factors of abdominal hernias among adults in Saudi Arabia.

## Materials and methods

Our study is a cross-sectional analytic study to measure the level of knowledge, awareness, and practice of ventral and inguinal hernias in Saudi Arabia. The data was collected by using a valid pretested structured questionnaire taken from previous studies after getting writing approval. The questionnaire included five parts: part one was designed to assess the demographic factors of the participants, including age, gender, educational level, residency, marital status, weight, and height. From weight and height, the Body Mass Index (BMI) of the participants was calculated, and the sample was classified as underweight, normal, overweight, and obese. The second part was used to assess the prevalence of hernia among the sample using the question “Have you had a hernia before?”. The third part was designed to assess the practice of the participants, including performing the exercise, having any congenital anomaly, having previous abdominal surgery, having a family history of hernia, having more than five pregnancies, having a history of prostatic enlargement, having asthma or chronic cough, and having chronic constipation. Moreover, knowledge was assessed depending on questions of part four using 14 questions, including 12 questions with yes and no answers and two questions to assess the knowledge considering treatment of choice and complications after surgical treatment. Part five was prepared to assess the participants’ views considering their awareness regarding hernia. After reviewing similar studies, the sample size is expected to be around 1000. We included any person more than 18 years who are willing to enroll in the study. Anyone who refuse to participate in the study has a mental and physical illness, is younger than 18 years, or didn’t answer the whole questionnaire was excluded.

We choose a small sample from the target population and conduct the survey. Data was analyzed afterward to ensure the clarity of the questionnaire and discover any shortages in the project plan. Data from the pilot study was not included in the final project analysis, and no changes were conducted to the questionnaire upon the pilot study.

All values were analyzed using the IBM Corp. Released 2019. IBM SPSS Statistics for Windows, Version 26.0. Armonk, NY: IBM Corp (for windows: evaluation version). In addition, P < 0.05 was considered statistically significant. Qualitative variables were expressed in the form of frequencies and percentages. The data were analyzed to test the level of knowledge, awareness, and practice towards hernia among the candidates.

We obtained ethical approval from the Qassim Research Committee with the reference number H-04-Q-001. The participants were informed about the purpose of the research, and we obtained electronic consent before completing the questionnaire. Data were identified initially and then coded in the database Microsoft Excel sheet (Redmond, USA) using a unique identification number. The data was stored on a password-protected laptop with the principal investigator (PI) and corresponding investigator (CI), and all data was maintained confidential. Only the research team had access to the database for analyses purpose. The manuscript included only the summary of the statistics without publication of the raw data, including the identifying information of the participants.

## Results

In this study, we were able to collect data from 2611 individuals in different regions of Saudi Arabia, including 27.9% from the western region, 25.2% from the central region, 20.9% from the southern region, 15.9% from the northern region, 10.1% from the southern region. Moreover, 68.5% of the participants were females, and 60.1% were aged between 18-29 years old. Furthermore, 73.2% of the participants studied till college, and 50.6% were single. Considering BMI, 44.9% of the participants had normal weight, while the prevalence of underweight, overweight, and obese were 9.7%, 26.5%, and 18.9%, respectively (Table 1).

**Table 1:** The demographic factors of the participants (N=2611)

		Count	Column N %
Gender	Male	822	31.5%
	Female	1789	68.5%
Age	18-29	1568	60.1%
	30-40	582	22.3%
	> 40	461	17.7%
Residency	Northern region	416	15.9%
	Southern region	546	20.9%
	Eastern region	263	10.1%
	Western region	729	27.9%
	Central region	657	25.2%
Education	Illiterate	10	0.4%
	Intermediate	106	4.1%
	Secondary	351	13.4%
	University graduate	1911	73.2%
	Postgraduate	233	8.9%
Marital status	Single	1322	50.6%
	Married	1216	46.6%
	Divorced	73	2.8%
BMI	Underweight	252	9.7%
	Normal weight	1166	44.9%
	Overweight	688	26.5%
	Obese	491	18.9%

**Figure 1:**
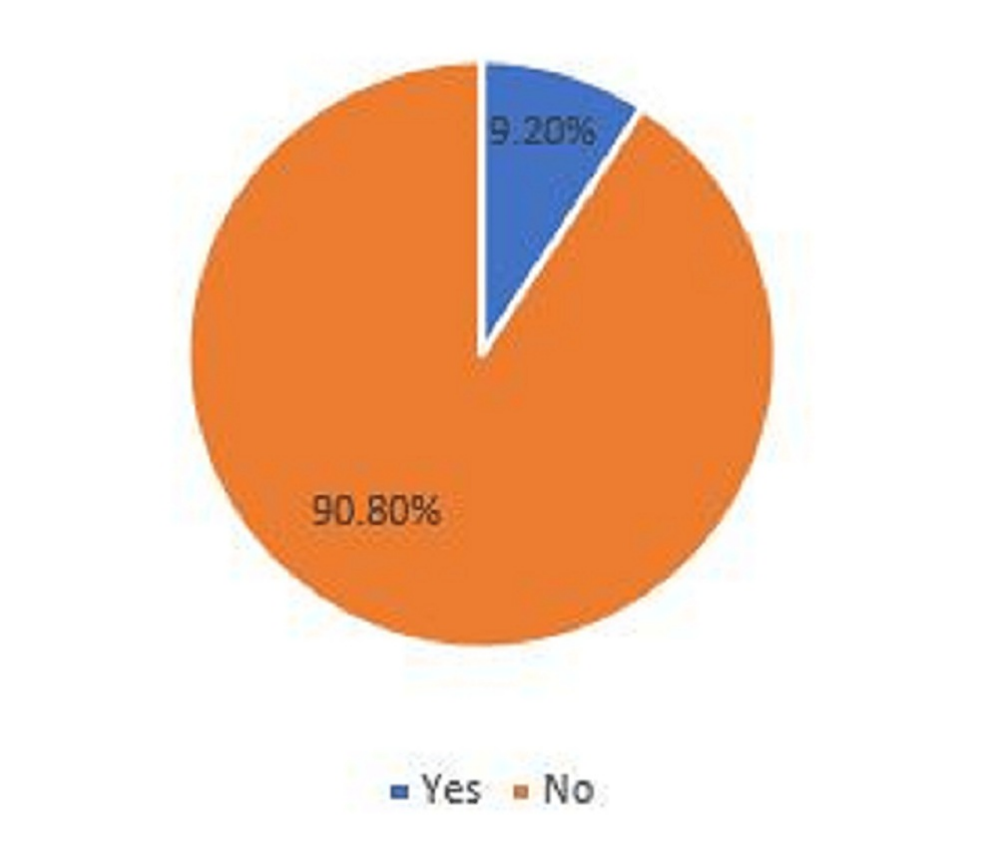
The prevalence of hernia, according to the participants

The prevalence of hernia among individuals in the current study was 9.2% (Figure 1). Moreover, we found that there is no significant difference between the two genders considering the prevalence of hernia (10.7% in males, and 8.5% in females, P=0.070). Furthermore, we found that the prevalence of hernia increases significantly with an increase in age, where the prevalence of hernia among participants older than 40 years old was 19.1% compared with 11.7% in the age group of 30-40 years and 5.4% in those with the age group of 18-29 (P<0.001). Moreover, the prevalence of hernia was the highest among participants in the northern region (16.6%), followed by those of the central region (11.6%), and the lowest was among individuals of the western region (4.5%) (P<0.001). Furthermore, the prevalence of hernia was the highest among illiterate individuals (30.0%), followed by postgraduate individuals (21%) and intermediate-educated individuals (20.8%) (P<0.001). Moreover, the prevalence of hernia was significantly lower in the single population (5.1%) compared with married (13.2%) and divorced (15.1%) (P<0.001). Considering the impact of BMI, we found that being overweight or obese increase the prevalence of hernia significantly (P<0.001), where the prevalence of hernia was 12.6% among obese participants, 10.9% in overweight ones, and 7.8% among normal-weight participants (Table 2).

**Table 2:** The relation between the prevalence of hernia and demographic factors

		Have you had a hernia before?				
		No		Yes		P-value
		Count	Row N %	Count	Row N %	
Gender	Male	734	89.3%	88	10.7%	0.070
	Female	1637	91.5%	152	8.5%	
Age	18-29	1484	94.6%	84	5.4%	0.000*
	30-40	514	88.3%	68	11.7%	
	> 40	373	80.9%	88	19.1%	
Residency	Northern region	347	83.4%	69	16.6%	0.000*
	Southern region	503	92.1%	43	7.9%	
	Eastern region	244	92.8%	19	7.2%	
	Western region	696	95.5%	33	4.5%	
	Central region	581	88.4%	76	11.6%	
Education	Illiterate	7	70.0%	3	30.0%	0.000*
	intermediate	84	79.2%	22	20.8%	
	Secondary	330	94.0%	21	6.0%	
	University graduate	1766	92.4%	145	7.6%	
	Postgraduate	184	79.0%	49	21.0%	
Marital status	Single	1254	94.9%	68	5.1%	0.000*
	Married	1055	86.8%	161	13.2%	
	Divorced	62	84.9%	11	15.1%	
BMI	Underweight	240	95.2%	12	4.8%	0.000*
	Normal weight	1075	92.2%	91	7.8%	
	Overweight	613	89.1%	75	10.9%	
	Obese	429	87.4%	62	12.6%	

**Figure 2:**
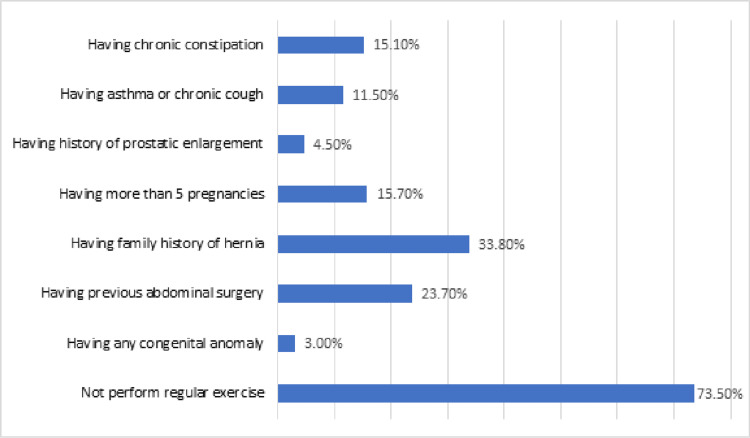
The prevalence of risk factors associated with hernia

Not performing regular exercise was the most common risk factor among participants toward hernia incidence, where 73.5% of the participants reported no performance of regular exercise, followed by having a family history of hernia (33.8%), having previous abdominal surgery (23.7%), and having more than five pregnancies in females (15.7%) (Figure 2).

The most popular known risk factors of hernia among the participants were lifting heavy things (83.8%), followed by weakness of the anterior abdominal wall (76.6%), pregnancy and labor (74.1%), previous abdominal surgery (67.9%) and obesity (65.8%) while congenital anomalies, diabetes mellitus and smoking were the least known risk factors only known by 25.4%, 27.6%, and 36.6%, respectively. 

Moreover, 48.1% of the participants thought that laparoscopic surgery is the treatment of choice for hernia, followed by open surgery (27.9%) and lifestyle modifications (20.3%). Furthermore, 15.5% of the participants thought that surgery had no complications, while post-operation pain/discomfort was the most known complication (54.5%), followed by recurrence of hernia (48.0%) and surgical site infection (45.1%). Moreover, 50.9% of the participants knew that surgical mesh is preferred for hernia repair. In general, 53.7% of the participants had a moderate level of knowledge, while 23.8% had a high level, and 22.5% had a low level of knowledge (Table 3).

**Table 3:** The knowledge of the participants toward hernia

		Count	N %
Do you think hernia has a relation to congenital anomalies?	No	1947	74.6%
	Yes	664	25.4%
Do you think weakness of the anterior abdominal wall increases the risk to develop hernia?	No	612	23.4%
	Yes	1999	76.6%
Do you think asthma or chronic cough increases the risk to develop hernia?	No	992	38.0%
	Yes	1619	62.0%
Do you think heavy lifting increases the risk to develop hernia?	No	422	16.2%
	Yes	2189	83.8%
Do you think chronic constipation increases the risk to develop hernia?	No	972	37.2%
	Yes	1639	62.8%
Do you think obesity increases the risk to develop hernia?	No	893	34.2%
	Yes	1718	65.8%
Do you think smoking increases the risk to develop hernia?	No	1656	63.4%
	Yes	955	36.6%
Do you think enlarged prostate increases the risk to develop hernia?	No	1536	58.8%
	Yes	1075	41.2%
Do you think pregnancy and labor increase the risk to develop hernia?	No	675	25.9%
	Yes	1936	74.1%
Do you think previous abdominal surgery increases the risk to develop hernia?	No	837	32.1%
	Yes	1774	67.9%
Do you think DM increases the risk to develop hernia?	No	1890	72.4%
	Yes	721	27.6%
What do you think is the treatment of choice for the hernia?	Laparoscopic surgery	1256	48.1%
	Open surgery	728	27.9%
	lifestyle modification	530	20.3%
	medical treatment	97	3.7%
Complications of surgery	None	405	15.5%
	Recurrence	1253	48.0%
	Surgical site infection	1177	45.1%
	Post- op pain/discomfort	1423	54.5%
Do you know that surgical mesh is preferred for hernia repair?	No	1281	49.1%
	Yes	1330	50.9%
Knowledge	Low	587	22.5 %
	Moderate	1402	53.7 %
	High	622	23.8 %

As recorded in Table 4, it was noticed that females had a higher level of knowledge than males significantly (P=0.001), where 27.1% of males had a low level of knowledge compared with 20.3% of females. Moreover, younger participants were found to have significantly better knowledge than older participants, where 29.2% of the participants in the age group of 18-29 years had high knowledge compared with 16.5% of the age group of 30-40 years and 14.8% of those older than 40 years (P<0.001). A higher level of education was significantly associated with a better level of knowledge, where 26.0% of university individuals had high knowledge compared with 17.1% of secondary school-educated individuals and 11.3 % of the intermediate-educated population (P<0.001). Moreover, singles showed the highest level of knowledge significantly (P<0.001). No significant difference was reported between individuals of different BMI (P=0.199). Moreover, participants who reported having a hernia showed a lower level of knowledge, where 13.8% of them had high knowledge compared with 24.8% of those who reported not having a hernia (P<0.001). Finally, those who self-reported excellent awareness reported a higher level of knowledge (55.7 %), 33.9% reported good awareness, 23.4% reported moderate awareness and 10.2% reported weak awareness (P<0.001) (Table 4).

**Table 4:** The relation between knowledge of the participants and demographic factors

		Knowledge						
		Low		Moderate		High		
		Count	N %	Count	N %	Count	N %	
Gender	Male	223	27.1%	415	50.5%	184	22.4%	0.001*
	Female	364	20.3%	987	55.2%	438	24.5%	
Age	18-29	354	22.6%	756	48.2%	458	29.2%	0.000*
	30-40	136	23.4%	350	60.1%	96	16.5%	
	> 40	97	21.0%	296	64.2%	68	14.8%	
Residency	Northern region	123	29.6%	231	55.5%	62	14.9%	0.000*
	Southern region	118	21.6%	266	48.7%	162	29.7%	
	Eastern region	56	21.3%	141	53.6%	66	25.1%	
	Western region	157	21.5%	365	50.1%	207	28.4%	
	Central region	133	20.2%	399	60.7%	125	19.0%	
Education	Illiterate	0	0.0%	9	90.0%	1	10.0%	0.000*
	intermediate	30	28.3%	64	60.4%	12	11.3%	
	Secondary	104	29.6%	187	53.3%	60	17.1%	
	University graduate	413	21.6%	1002	52.4%	496	26.0%	
	Post graduate	40	17.2%	140	60.1%	53	22.7%	
Marital status	Single	285	21.6%	636	48.1%	401	30.3%	0.000*
	Married	280	23.0%	732	60.2%	204	16.8%	
	Divorced	22	30.1%	34	46.6%	17	23.3%	
BMI	Underweight	57	22.6%	120	47.6%	75	29.8%	0.199
	Normal weight	263	22.6%	622	53.3%	281	24.1%	
	Overweight	149	21.7%	380	55.2%	159	23.1%	
	Obese	110	22.4%	278	56.6%	103	21.0%	
Have you had a hernia before?	No	534	22.5%	1248	52.6%	589	24.8%	0.000*
	Yes	53	22.1%	154	64.2%	33	13.8%	
Your awareness level regarding hernia	Week	256	30.2%	506	59.6%	87	10.2%	0.000*
	Moderate	232	22.1%	574	54.6%	246	23.4%	
	Good	69	14.1%	254	51.9%	166	33.9%	
	Excellent	30	13.6%	68	30.8%	123	55.7%	

## Discussion

The abdominal hernia and its procedures are a common complaint between both males and females, particularly the umbilical and para-umbilical hernia [3]. The current study aimed to know the prevalence of hernias in Saudi Arabia and to assess the awareness of the risk factors of abdominal hernias among adults in Saudi Arabia.

In the current study, the prevalence of hernia was 9.2% which is similar to the results of some previous studies, including the study of Kibert et al., which reported that the prevalence of hernia among the public population in northern Ethiopia was 11.7% [4], the study of Alenazi A et al., which reported that the prevalence of hernia among the public population in Arar city, Saudi Arabia was 11.5% [5]. However, our prevalence was higher than reported in other studies conducted in Egypt, Ghana, and Uganda, which reported a prevalence of hernia between 3.4% and 8.1% [6-8]. The current study did not find a significant difference in the prevalence of hernia between males and females; however, the prevalence of hernia was slightly higher in males. This is not consistent with the results of Alenazi A et al., who reported that the prevalence of hernia was significantly higher among females than males (63.4% vs. 36.6%) [5], and the study of Bedewi et al., who reported that para-umbilical hernia positive cases among females were 24.9% which is slightly higher than males (23.3%) [9]. Moreover, our results indicate that older age participants were more significantly likely to be diagnosed with external hernia compared with younger participants. These results are supported by the results of different studies elsewhere [4,10-12]. The reason associated with that result could be attributed to the fact of the degenerative weakness of abdominal muscles and fibrous tissue reported in the elderly age group, where the loss of abdominal muscle strength and resistance to high intra-abdominal pressure could be associated with increased risk of herniation [10,13-15]. Moreover, the decline in blood testosterone level and increase in estrogen level via the action of the aromatase enzyme in older participants is another potential reason for increasing the prevalence of hernia among older participants where lower abdominal muscles are sensitive to the body’s estrogen hormone and tend to express a very high level of estrogen receptor; and thus, the increase in the estrogen level could lead to atrophy and fibrosis of lower abdominal muscles which results in increasing the occurrence in older participants, especially among males [16]. Moreover, obesity and being overweight are other risk factors for the increased prevalence of hernia, which is similar to the results of many previous studies [13-16]. In the current study, not performing regular exercise was the most common risk factor among participants toward hernia incidence, where 73.5% of the participants reported no performance of regular exercise, followed by having a family history of hernia (33.8%), having previous abdominal surgery (23.7%), and having more than five pregnancies in females (15.7%).

Considering the knowledge, we found that 83.8% of the participants correlated between hernia and lifting heavy things, while 76.6% correlated it with weakness of the anterior abdominal wall, 74.1% with pregnancy and labor, 67.9% with previous abdominal surgery, and 65.8% with obesity while congenital anomalies, diabetes mellitus, and smoking were the least known risk factors only known by 25.4%, 27.6%, and 36.6%, respectively. In a previous study conducted by Alkhars A et al. among adults in Riyadh, Saudi Arabia, the authors found that the majority of participants (87%) suggested a correlation between hernia and heavy lifting, and 65% and 62% of the participants related pregnancy and surgery as a contributing factor for a hernia while lack of awareness was obvious on other risk factors such as smoking, chronic, constipation prostate enlargement, asthma and DM [17]. In another study conducted by Assakran et al., the authors reported that 83.9% of the participants knew that lifting heavy weights is a risk factor for developing a hernia, while 16.6% of them knew that diabetes and asthma are risk factors for hernia [18]. 48.1% of the participants in the current study thought that laparoscopic surgery is the treatment of choice for the treatment of hernia, followed by open surgery (27.9%) and lifestyle modifications (20.3%). Moreover, 50.9% of the participants thought that mesh repair is better to be used in hernia surgery which is similar to the results of the previous study [15]. According to a previous systematic review, it was found that depending on mesh repair was associated with a 50%-75% lower risk for hernia recurrence, an earlier return to work, and a lower risk for chronic post-herniorrhaphy groin pain than non-mesh repair [19]. In general, most of the participants in the current study had a moderate level of knowledge considering hernia. In a previous study, the authors showed that 50.9% of Saudis had good knowledge considering hernia [15].

In the current study, females, younger age, individuals with higher level of education, singles, and people who already have hernia had better knowledge about hernia. This is similar to the results of Mahfouz et al., who found that younger age, being single, and having a higher level of education was associated with better knowledge [15]. However, some other studies showed that herniation knowledge did not seem to differ at the gender level for all of the items of the research questionnaire [15-17,20]. Moreover, some previous studies showed that younger ages (18-28 years old) presented a higher level of hernia knowledge than other age groups [18,21], similar to our results. Many other studies reported similar results of hernia knowledge among the higher educated population [15,17,20,22].

This study had some limitations, including depending on a self-reported question to assess the prevalence of hernia, which may be associated with some personal bias where some persons may over- or underestimate their condition. More investigations depending on clinical diagnosis and hospital-based data will be more accurate in determining the prevalence of hernia. The dependence on a self-reported questionnaire may be associated with some random answers by some participants. 

## Conclusions

In conclusion, we found a moderate level of knowledge among adult individuals in Saudi Arabia about hernia. Being female, younger participants, having higher education, being single, already having hernia, and having good/excellent self-reported awareness were associated with a better level of knowledge. The prevalence of hernia was similar to those reported in previous studies; however, there is a higher incidence of risk factors in the current population. Older individuals, those who lived in the Northern region, being married, overweight and obese individuals were at a higher risk for developing a hernia. Not performing regular exercise, having a family history of hernia, and having previous abdominal surgery were the most common risk factors for hernia reported among individuals in the current study. There is a significant relationship between knowledge considering risk factors of hernia and the prevalence of hernia, indicating that there is a need to increase the knowledge and awareness of the public toward risk factors of hernia, which will be useful in reducing the prevalence of hernia in Saudi Arabia. Further studies should aim to confirm the association between the knowledge and prevalence of hernia among populations.
